# Faba Greens, Globe Artichoke’s Offshoots, Crenate Broomrape and Summer Squash Greens: Unconventional Vegetables of Puglia (Southern Italy) With Good Quality Traits

**DOI:** 10.3389/fpls.2018.00378

**Published:** 2018-03-27

**Authors:** Massimiliano Renna, Angelo Signore, Vito M. Paradiso, Pietro Santamaria

**Affiliations:** ^1^Department of Agricultural and Environmental Science, University of Bari Aldo Moro, Bari, Italy; ^2^Department of Soil, Plant and Food Science, University of Bari Aldo Moro, Bari, Italy

**Keywords:** by-products, culinary use, ethnobotany, landrace, nutritional value, traditional agri-food products

## Abstract

Globe artichoke (*Cynara cardunculus* L. subsp. [L.] *scolymus* Hayek), summer squash (*Cucurbita pepo* L.) and faba bean (*Vicia faba* L.) are widely cultivated for their immature inflorescences, fruits and seeds, respectively. Nevertheless, in some areas of Puglia (Southern Italy), other organs of these species are traditionally used as vegetables, instead of being considered as by-products. Offshoots (so-called *cardoni* or *carducci*) of globe artichoke, produced during the vegetative growing cycle and removed by common cultural procedures, are used like to the cultivated cardoons (*C. cardunculus* L. var. *altilis* DC). The stems, petioles, flowers and smaller leaves of summer squash are used as greens (so-called *cime di zucchini*), like other leafy vegetables such as chicory (*Cichorium intybus* L.) and Swiss chard (*Beta vulgaris* L.). Also the plant apex of faba bean, about 5–10 cm long, obtained from the green pruning, are used as greens (so-called *cime di fava*) like spinach leaves. Moreover, crenate broomrape (*Orobanche crenata* Forssk.), a root parasite plant that produces devastating effects on many crops (mostly legumes), is used like asparagus (*Asparagus officinalis* L.) to prepare several traditional dishes. In this study ethnobotanical surveys and quality assessment of these unconventional vegetables were performed. For their content of fiber, offshoots of globe artichokes can be considered a useful food to bowel. Summer squash greens could be recommended as a vegetable to use especially in the case of hypoglycemic diets considering both content and composition of their carbohydrates. For their low content of nitrate, faba greens could be recommended as a substitute of nitrate-rich leafy vegetables. Crenate broomrape shows a high antioxidant activity and may be considered as a very nutritious agri-food product. Overall, the results of the present study indicate that offshoots of globe artichoke, summer squash greens, faba greens and crenate broomrape have good potential as novel foods, being nutritious and refined products. Their exploitation aiming to the obtainment of labeled and/or new potential ready-to-eat retail products could satisfy the demand for local functional foods.

## Introduction

Puglia region (Southern Italy), placed in the center of the Mediterranean basin, has a long tradition in vegetable crops. It is very rich in landraces obtained by farmers themselves through repeated simple selection procedures generation after generation (Elia and Santamaria, 2013). Thus, many of these landraces are listed as an item in the ‘List of Traditional Agri-Food Products’ of the Italian Ministry of Agricultural, Food and Forestry Policies, since their processing, preservation and aging methods are consolidated in time, harmonious, according to traditional rules, for a period not less than 25 years. It is interesting to highlight that in Puglia region a relevant number of vegetable-based traditional dishes may be found. Therefore, several landraces of vegetables, for which there is a strong link with the regional traditions, are used as ingredients for preparing several dishes of the Puglia’s cuisine (Renna et al., 2015a).

The landrace vegetables of Puglia are appreciated both as refined food and for the intake of several healthy nutrients. For example, fruits of *Carosello* and *Barattiere* (herbaceous plants belonging to *Cucumis melo* L. species) are consumed at the immature stage, instead of cucumbers, not only for their organoleptic traits but also for the high potassium content and low amount of sodium and sugars (Serio et al., 2005). Polignano Carrot (a multicolored landrace of *Daucus carota* L., locally so-called *Carota di Polignano*) is greatly appreciated by people for its special taste, texture, flavor, fragrance and great variety of colors, that range from yellow to dark purple in the outer core and from pale yellow to light green in the inner core (Cefola et al., 2012; Renna et al., 2014b). Moreover, for its high antioxidant activity as well as for its high content in total phenols, carotenoids and β-carotene it can be regarded also as a functional food (Cefola et al., 2012; Renna et al., 2014b). *Galatina* and *Molfettese* stem chicories (two landraces of *Cichorium intybus* L., Catalogna group), useded both raw and cooked, represent a refined and nutritious vegetables, because of the presence of several healthy compounds as well as their low nitrate content (Renna et al., 2014a; Testone et al., 2016; D’Acunzo et al., 2017).

**FIGURE 1:**
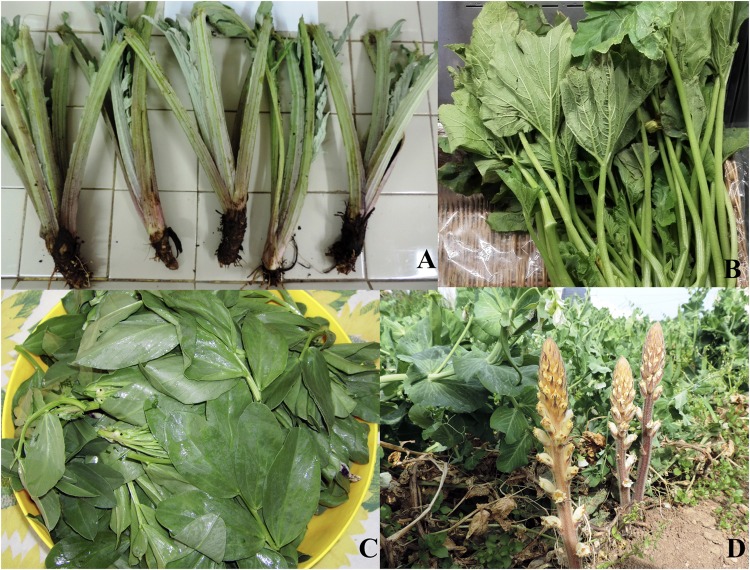
Offshoots removed from plants of globe artichoke **(A)**. Summer squash greens **(B)**. Faba greens **(C)**. Crenate broomrape **(D)**.

An interesting and time-honored custom of Puglia counts the culinary use of several plant parts, which can be considered as “unconventional” agri-food products. This because for a few vegetable species some plant parts are usually treated as by-products or wastes of the agri-food chain and not as a food. For example, globe artichoke (*Cynara cardunculus* L. subsp. [L.] *scolymu*s Hayek) is widely cultivated for its immature inflorescences which can be considered a very important food product of the Mediterranean diet. Plants of globe artichoke are generally propagated vegetatively by offshoots (**Figure 1A**) which are continuously produced during the vegetative cycle. Thus, a part of them is removed from the field by common cultural procedures. These offshoots (**Figure 1A**) are usually considered as by-products, but in some areas of Puglia (Southern Italy) they are traditionally used as a food ingredient, like to the cultivated cardoons (*C. cardunculus* L. var. *altilis* DC). Summer squash (*Cucurbita pepo* L.) is widely cultivated in the world for both its fruits and flowers. Nevertheless, in some areas of Puglia region its stems, petioles and smaller leaves are used as “greens” (**Figure 1B**), like other leafy vegetables such as chicory (*Cichorium intybus* L.) and Swiss chard (*Beta vulgaris* L.). The faba bean (*Vicia faba* L.) is widely cultivated in the world for its seed, especially as a dry legume. In some areas of Puglia the plant apex of faba bean, about 5–10 cm long, obtained from the green pruning, are used as “greens” (**Figure 1C**) like spinach leaves (*Spinacia oleracea* L.). Finally, crenate broomrape (*Orobanche crenata* Forssk.), a parasite plant (**Figure 1D**) that produces devastating effects on many crop (mostly legumes). In a few areas of Puglia this parasite is considered a wild edible plant used, like asparagus (*Asparagus officinalis* L.), for preparing several traditional dishes (Renna et al., 2015a,b).

The culinary use of these products in Puglia, like several wild edible plants, has ancient origins and is due to the food scarcity and poverty of the ancestors in the past. Thus, instead of being eliminated as by-products, the culinary use of these plant parts enabled farmers to gain a precious extra food source (Bianco et al., 1998; Elia and Santamaria, 2013).

Nowadays these unconventional vegetables could satisfy the needs of specific markets. This because niche food products, founded on quality and agricultural biodiversity and intended for local use, seems to meet requirements of consumers about safety and genuineness. Therefore, it is of high importance to evaluate the nutritional traits of these products for further exploitation activities aimed to produce high added value products and by reinforcing local rural economies. Moreover, especially for specific niche markets, characterized by a high demand of local products grown through sustainable techniques, it is essential to disseminate the knowledge about landraces (Signore et al., 2014). At the same time, also local traditions and cultural identity of people could be preserved and promoted, assuming that each product is intimately linked to the local identity, with specific geographic, climatic, environmental and cultural characteristics (Renna, 2015).

To the best of our knowledge, there is a lack of information in literature with regard to ethnobotanical information as well as to the nutritional characterization of these unconventional vegetables obtained from Puglia landraces. Starting from these considerations, a compositional analysis of globe artichoke’s offshoots, faba greens, summer squash greens and crenate broomrape was performed. The general goal was to assess some quality traits, such as proteins, lipids, total carbohydrates, fiber, mineral content and antioxidant activity, in order to furnish an overview and evaluate the potential of these foodstuffs as traditional agri-food products for culinary uses.

## Materials and Methods

### Study Site and Ethnobotanical Surveys

**FIGURE 2:**
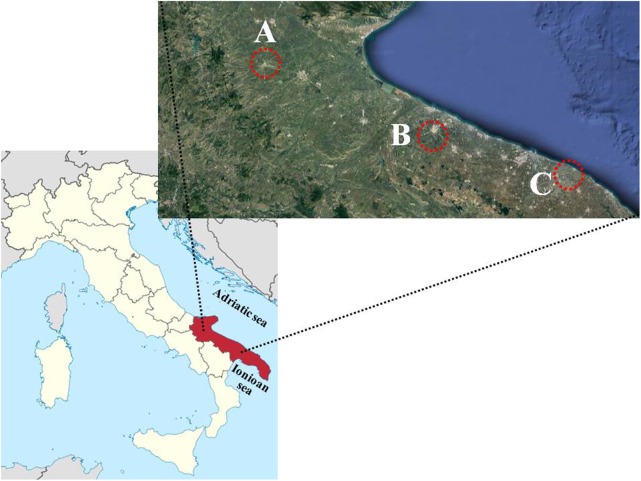
Map of Italy with Puglia Region in red and the areas (within the circle) where offshoots of globe artichokes and summer squash greens were grown: Lucera **(A)**; Andria **(B)**; Mola di Bari **(C)**.

Puglia region (**Figure 2**), 350 km long and 60 km narrow, is largely open to the Adriatic and Ionian seas with a coastal zone of nearly 800 km. Its area of about 19,360 km^2^ shows more than 60% of territory below 200 m above sea level, with some peaks of more than 1000 m located in the North-East and North-West. The climate is semi-arid Mediterranean, characterized by high temperatures and drought in summer and moderately cold temperatures and rainfall in winter. Thanks to its geographical length and variety of orographic and pedoclimatic conditions, Puglia region shows an interesting richness of vegetables crop.

Ethnobotanical studies on landraces cultivated by seed savers were conducted though field studies and *ad hoc* investigations about the culinary customs and folk use of vegetable landrace. Additionally, ethnobotanical papers as well as folkloric and gastronomic literature, were also considered.

### Plant Material and Sample Preparation

Four different types of unconventional vegetables were collected and analyzed: globe artichoke’s offshoots, faba greens, summer squash greens and crenate broomrape. Offshoots were removed for plants of two different globe artichoke landrace: *Carciofo di Lucera* (late genotype) and *Locale di Mola* (early genotype) (**Figure 2**). Summer squash greens and faba greens were collected from plants of landraces of Andria and Mola di Bari, respectively, (**Figure 2**). Crenate broomrape was collected from yield cultivated with different landraces of faba bean. Immediately after harvesting, samples of each type of unconventional vegetable were refrigerated and then transferred to the laboratory to be processed and analyzed. After removing inedible parts, samples were washed with tap water, blotted dry with paper towels and cut to obtain edible portions of vegetables. Each replicate was freeze-dried (ScanVac CoolSafe 55-9 Pro; LaboGene ApS, Lynge, Denmark) and then packed in hermetic jars and stored in the dark at -21 ± 1°C until the analyses were carried out.

### Chemical Analysis

Proximate analysis of samples was carried out as follows: ashes were determined by muffle furnace according to AOAC method 923.03 (AOAC, 1991); proteins content (*N* × 6.25) was determined by Kjeldahl nitrogen according to AOAC method 955.04 (AOAC, 1991); fat content was determined by Soxhlet extraction according to AOAC method 920.39 (AOAC, 1991); dietary fiber content was determined by enzymatic-gravimetric procedure according to AOAC method 991.43 (AOAC, 1991); moisture content by automatic moisture analyzer (Mod. MAC 110/NP, Radwag Wagi Elektroniczne, Radom, Poland) at 105°C; and total carbohydrates were calculated by difference of protein, lipid and ash on the dry matter basis.

Before inulin extraction, globe artichoke’s offshoots were subdivided into root, external leaves and edible parts. Inulin was extracted from globe artichoke’s offshoots according to Ronkart et al. (2007) with some adaptations. 250 grams of minced sample were extracted with 800 mL of hot water (80°C) for 90 min. The pH of water was adjusted to 6.8 with NaOH in order to avoid inulin hydrolysis at pH < 6. Extracted juice was filtered on a Büchner funnel with 11 μm Whatman filters N. 1. Inulin was then precipitated by two cycles of freezing/thawing followed by centrifugation at 7500 × *g* and 10°C for 15 min. The pellet was washed with 10 mL of acetone, centrifuged, dried and weighted.

Antioxidant activity was determined by the 2,2-diphenyl-1-picrylhydrazyl (DPPH) stable radical scavenging capacity test, according to Difonzo et al. (2017). Samples (0.1 g) were extracted with 5 mL water:methanol (80:20) for 2 h in tubes covered with aluminum foil. Extracts were then centrifuged for 15 min at 15000 *g* and 24°C. The supernatant was recovered and filtered with PTFE septa (0.45 μm). Extracts (50 μL) were added to 950 μL of 0.08 mM DPPH in ethanol. The mixture was shaken and left at room temperature in the dark for 30 min. The decrease of the absorbance at 517 nm was measured using a Cary 60 Agilent spectrophotometer (Agilent Technologies, Milan, Italy). The results were expressed in μmol Trolox equivalents (TE) g^-1^ dry weight. Each sample was analyzed in triplicate.

For inorganic ion content an ion exchange chromatography (Dionex DX120; Dionex Corporation, Sunnyvale, CA, United States) with a conductivity detector was performed as reported by D’Imperio et al. (2016). Nitrate (NO_3_^-^) content was determined in 0.5 g dried sample using an IonPac AG14 precolumn and an IonPac AS14 separation column (Dionex Corporation). Cation contents (Na^+^, K^+^, Mg^2+^, and Ca^2+^), were determined in 1 g dried sample using an IonPac CG12A guard column and an IonPac CS12A analytical column (Dionex Corporation). In order to determine glucose, fructose and saccharose contents, samples were prepared by protocols used by Renna et al. (2014a) and analyzed by ion chromatography (Dionex DX-500; Dionex Corp., Sunnyvale, CA, United States) using a pulsed amperometric detector. Peak separation was performed using a Dionex CarboPac PA1 and isocratic elution with 50 mmol L^-1^ NaOH.

### Experimental Design and Data Analysis

Three series for each unconventional vegetable were harvested in order to provide independent replicates. For each replicate, all samples were then mixed well in order to obtain a bulk sample. Chemical analyses were performed in triplicate for each bulk sample. To detect statistical significance, ANOVA was applied (GLM procedure, SAS software) and means were separated by the Student–Newman–Keuls (SNK) test.

## Results

### Ethnobotanical Information

**FIGURE 3:**
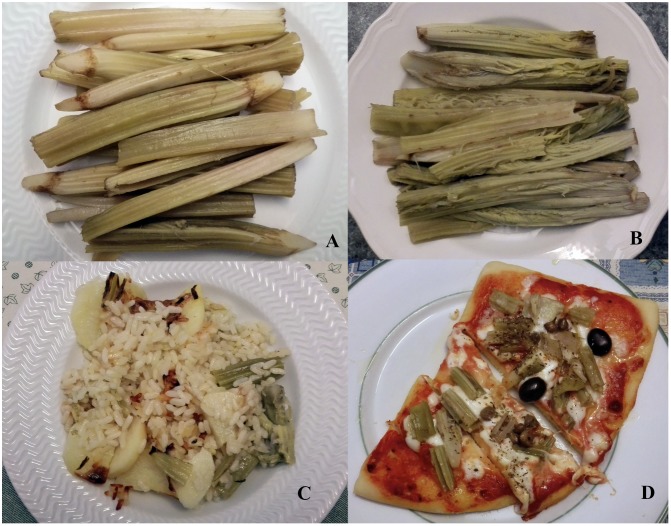
Dishes based on offshoots of globe artichoke: boiled *Locale di Mola* **(A)** and *Lucera* **(B)** landraces; baked potatoes, rice and offshoots **(C)**; pizza with tomato sauce, mozzarella cheese, olives, capers and offshoots **(D)**.

During the first half of the 20th century, some authors (Gramignani, 1934; Tamaro, 1937; Turchi, 1957) have described the techniques to be applied to artichoke plants for producing tender and light colored offshoots, so-called “*gobbi*” (hunchbacked) because they were curved to the soil. In Italian language the offshoots of globe artichoke are named *cardoni* or *carducci*. It is likely that these local names are due to the similarity of the globe artichoke’s offshoots to both cultivated and wild cardoons. It is also interesting to note that in Puglia about 300 families with the name “*Cardone*” can be found, while in Italy over 1,300 families with this name can be found (Renna and Santamaria, 2017). Before being used as a food ingredients, fibrous parts of the offshoots must be eliminated and, after boiling, a soaking process in water for some hours is need for reducing their bitter taste (Renna et al., 2015a). In 2016, the offshoots of globe artichoke have been listed as an item in the ‘List of Traditional Agri-Food Product of Puglia’ of the Italian Ministry of Agricultural, Food and Forestry Policies, because of their consolidated culinary use in the region for over 25 years. Indeed, in Puglia it is well known the home-made culinary preparation of this unconventional vegetable. Moreover, some gastronomic books of traditional cuisine reported several recipes, such as first courses, side dishes, main courses and pizza with using the offshoots of globe artichokes (**Figure 3**) similar to recipes based on cardoons and heads of globe artichoke (Sada, 1991).

**FIGURE 4:**
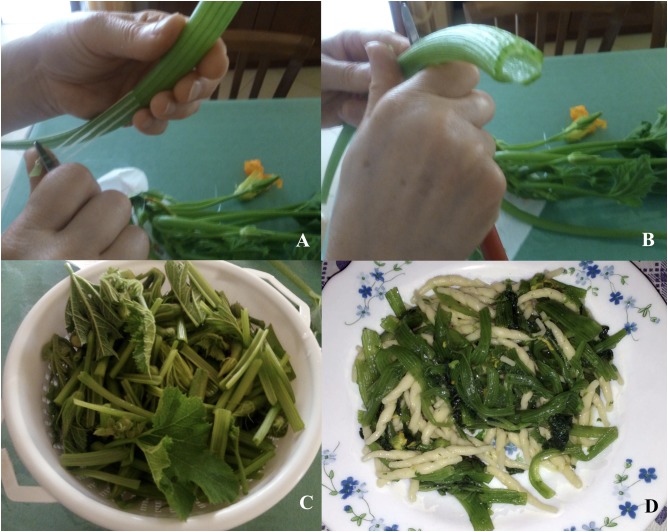
Home-made culinary preparation of summer squash greens: removal of fibrous filaments **(A)**; cutting **(B)**; edible parts **(C)**; pasta with summer squash greens **(D)**.

Summer squash greens are traditionally used as a food almost exclusively in some territories of the Southern Italy, especially in Puglia. In Italian summer squash greens are named *cime di zucchina*. Most likely, the culinary use of this vegetable originated from the peasantry cuisine of these territories. Effectively, stems, petioles and smaller leaves of the summer squash are one of the main ingredients of the so-called “survival cuisine”, which in periods of poverty has constrained to exploit any edible part of the vegetable plants (Renna and Santamaria, 2017). Therefore, toward the end of the productive cycle, our ancestor harvested stems, petioles and smaller leaves in order to better exploit all parts of the summer squash plant. Moreover, in the past, farmers used as summer squash greens also the excess seedlings, so-called *siverchi*, that were removed from the field as a consequence of overabundant use of seeds during sowing respect to the optimal plant density in the field (Renna and Santamaria, 2017). The first known information regarding the culinary use of summer squash greens is found in an ancient book dating back to 1576 (Ingrassia, 1576). In this book, the author has described the food use of the summer squash greens between some good nutrition practices useful for preventing the plague contagion that in those times was much feared. Moreover, summer squash greens were also described as a food ingredient in another ancient book dating back to 1824 (Soderini, 1824). Nowadays, summer squash greens are traditionally used especially with the pasta for preparing first courses but also for preparing side dishes like for other conventional leafy vegetables such as chicory and Swiss chard. Anyway, before to use as food ingredient it is important to remove all fibrous filaments from stems and petioles of the summer squash greens (**Figure 4**).

**FIGURE 5:**
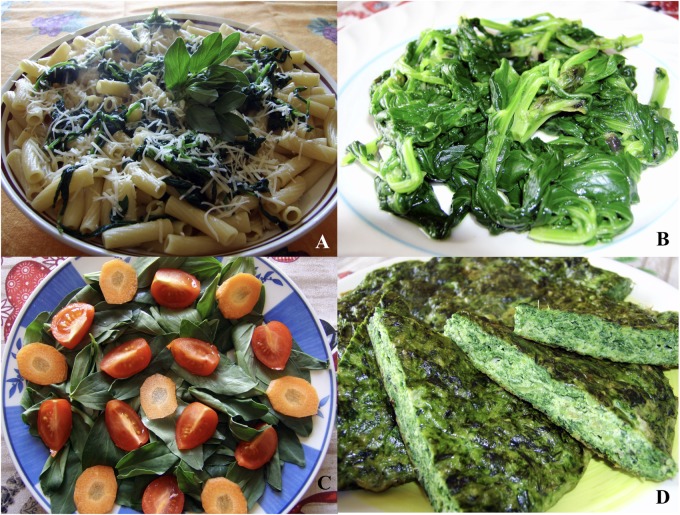
Dishes based on faba greens: first course pasta-based **(A)**; boiled **(B)**; salad with cherry tomatoes and carrots **(C)**; baked omelet **(D)**.

In Italian faba greens are named *cime di fava*. The green pruning of the faba plant apex is an ancient operation that farmers carried out to satisfy several cultural needs such as the deterrent action against aphids. It is not a case that this specific action is well described by Margaroli in an ancient book dating back to 1831 (Margaroli, 1831). According to folk knowledge, in the past the plant apexes removed by means of pruning was not considered as a by-product but as a springy vegetable. Indeed, faba greens can be eaten raw, for example in salads, or cooked like spinach to be used in pasta dishes or into quiches and omelets (**Figure 5**). It is likely that the similarity of the faba greens to spinach leaves (**Figure 1C**) was a decisive factor in using this part of the plant as a food. Nevertheless, taste, smell and texture of the faba greens are very different from ones of spinach leaves.

**FIGURE 6:**
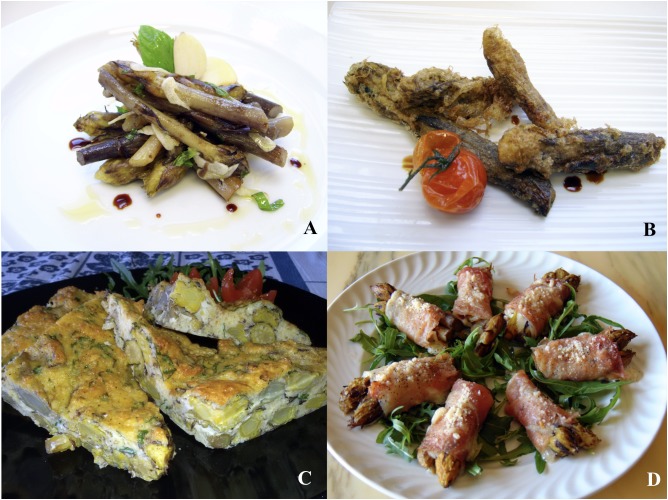
Dishes based on crenate broomrape: salad with garlic, mint, vinegar and extra virgin olive oil **(A)**; floured and fried **(B)**; vegetable pie **(C)**; gratineéd ham roll **(D)**.

The earliest known information on crenate broomrape as a food is found in Pliny the Elder’s Book of Natural History: … *it is a small leafless stem that can be eaten*… (Bianco et al., 2009). Similarly, the culinary use of crenate broomrape was described in an ancient book of the 15th century (Bianco et al., 2009). Nevertheless, several authors have been reported negative information describing this parasitic plant. For example, Matthioli (1613) and Redi (Bianco et al., 2009) called broomrape “wolf herb” on account of its ability to “eat up” adjacent plants (Bianco et al., 2009). In some areas of Puglia the term s*porchia* is one of the common local names used for *O. crenata* L.. It is interesting to underline that the etymology of this local name, is due to the very high quantity of seeds produced by this parasitic plant and descends from the Latin “*exporculare*” (Bianco et al., 2009). Crenate broomrape was a real disaster for the main legume crops, therefore its elimination from the fields allowed to preserve an important crop and obtain an extra food product. It is not a case that its culinary use is reported in some books as well as in ethnobotanical and scientific papers (Bianco et al., 1998, 2009; Perrino et al., 2004; Renna et al., 2015a,b). In some areas of Puglia, crenate broomrape is widely used for several traditional dishes. Probably, its resemblance to large asparagus was a decisive factor in harvesting and consuming this parasitic plant. Stems of crenate broomrape are cleaned, washed and boiled. In a similar way for offshoots of globe artichoke, also for crenate broomrape a soaking process in water (12–24 h) is needed for reducing their bitter taste (**Figure 6**). Today. the culinary use of crenate broomrape is a part of the traditional Puglia’s cuisine with a linkage to the Mediterranean Diet (Renna et al., 2015a). In 2015 crenate broomrape has been listed as an item in the ‘List of Traditional Agri-Food Product of Puglia’. It is interesting to highlight that nowadays local farmers harvest and sell crenate broomrape for up to three times as much as fresh broad beans (Renna et al., 2015b).

### Nutritional Traits

**Table 1** shows the proximate composition of globe artichoke’s offshoots, summer squash greens, faba greens and crenate broomrape. The highest water content was found in summer squash greens, followed by *Locale di Mola* and *Lucera* globe artichoke’s offshoots, while the lowest content was found in both faba greens and crenate broomrape (about 85 g 100 g^-1^ fresh weight - FW - on average). As regard the protein content the highest value was found in faba greens. Summer squash greens showed a content of protein about 75% lower respect to faba greens and about 40% lower respect both crenate broomrape and offshoots of *Lucera* globe artichoke. At the same time, no significant differences about protein content were found between offshoots of *Locale di Mola* globe artichoke and summer squash greens (**Table 1**). Crenate broomrape showed the highest content of both total lipid and carbohydrate, while in summer squash greens was found the lowest content of these food components (**Table 1**). It is interesting to highlight that the carbohydrate content in crenate broomrape was higher of about 32, 78, 114, and 281% compared with faba greens, offshoots of *Lucera* globe artichoke, offshoots of *Locale di Mola* globe artichoke and summer squash greens, respectively (**Table 1**). With respect the dietary fiber, offshoots of *Lucera* globe artichoke showed a higher content respect both summer squash greens and crenate broomrape (**Table 1**). Moreover, no significant differences were found between faba greens and offshoots of *Locale di Mola* globe artichoke also as compared with all other unconventional vegetables. Finally, ashes content was highest in both offshoots of *Lucera* globe artichoke and summer squash greens, and lowest in faba greens, crenate broomrape and offshoots of *Locale di Mola* globe artichoke.

**Table 1:** Proximate composition of globe artichoke’s offshoots, summer squash greens, faba greens and crenate broomrape.

	Globe artichoke’s offshoots		Summer squash greens	Faba greens	Crenate broomrape	Significance
					
	Lucera	Locale di Mola				
Water	89.38c	91.48b	94.06a	84.77d	85.20d	^∗∗∗^
Protein	2.41b	1.82bc	1.36c	5.35a	2.09b	^∗∗∗^
Total lipid	0.30b	0.22b	0.06c	0.21b	0.40a	^∗∗∗^
Total carbohydrate	6.44c	5.36c	3.01d	8.68b	11.46a	^∗∗∗^
Fiber, total dietary	4.03a	3.14ab	1.88b	3.15ab	2.20b	^∗∗∗^
Ashes	1.47a	1.11b	1.50a	1.00b	0.86b	^∗∗∗^


*Mean values are expressed as g 100 g^-*1*^ FW. For each parameter, the same letters in the same row indicate that mean values are not significantly different (*P* = 0.05). Significance: ^∗∗∗^*P* ≤ 0.001.*

In globe artichoke’s offshoots the mean value of the inulin content was of 1.04 g 100 g^-1^ FW and 1.41 g 100 g^-1^ FW, respectively, for *Lucera* and *Locale di Mola* landraces.

The content of principal cations and nitrate in globe artichoke’s offshoots, summer squash greens, faba greens and crenate broomrape is reported in **Table 2**. The highest content of sodium was found in offshoots of *Locale di Mola* globe artichoke, while the lowest content was found in summer squash greens, faba greens and crenate broomrape (about 19.7 mg 100 g^-1^ FW on average). Offshoots of *Lucera* globe artichoke showed a sodium content about 70% lower compared with offshoots of *Locale di Mola* globe artichoke, but about 2.4-fold higher compared with summer squash greens, faba greens and crenate broomrape (**Table 2**). Potassium content was highest in both summer squash greens and offshoots of *Locale di Mola* globe artichoke (about 489 mg 100 g^-1^ FW on average), followed by faba greens and both crenate broomrape and offshoots of *Lucera* globe artichoke that showed the lowest values. As regards magnesium, summer squash greens showed a content about 54% higher compared with crenate broomrape, while no significant differences were found respect to other unconventional vegetables (**Table 2**). The highest content of calcium was found in both types of globe artichoke’s offshoots and summer squash greens (about 99 mg 100 g^-1^ FW on average), while the lowest content was found in both faba greens and crenate broomrape (about 57 mg 100 g^-1^ FW on average). As regards nitrates, the lowest content was found in summer squash greens. Faba greens and offshoots of *Lucera* globe artichoke showed a nitrate content (about 97 mg 100 g^-1^ FW on average) about 7.4-fold higher compared with summer squash greens and about 90% higher compared with crenate broomrape, while no significant differences were found between faba greens and both type of globe artichoke’s offshoots (**Table 2**).

**Table 2:** Content of principal cations, nitrate and sugars in globe artichoke’s offshoots, summer squash greens, faba greens and crenate broomrape.

	Globe artichoke’s offshoots		Summer squash greens	Faba greens	Crenate broomrape	Significance
					
	Lucera	Locale di Mola				
Na^+^	48.11b	159.52a	16.89c	21.35c	20.87c	^∗∗∗^
K^+^	489.76a	184.63c	488.88a	323.95b	252.95c	^∗∗∗^
Mg^2+^	29.23ab	27.68ab	38.41a	32.99ab	24.98b	^∗^
Ca^2+^	107.46a	91.31a	97.91a	63.33b	51.18b	^∗∗∗^
NO_3_^-^	110.42a	86.11ab	13.03c	82.86a	50.92b	^∗∗∗^
Glucose	767.4a	815.0a	107.1c	186.3c	477.3b	^∗∗∗^
Fructose	315.3b	175.2c	134.3c	112.1c	995.2a	^∗∗∗^
Sucrose	197.7b	89.0b	37.5b	446.6a	190.8b	^∗∗^


*Mean values are expressed as mg 100 g^-*1*^ FW. For each parameter, the same letters in the same row indicate that mean values are not significantly different (*P* = 0.05). Significance: ^∗∗∗^*P* ≤ 0.001; ^∗∗^*P* ≤ 0.01; ^∗^*P* ≤ 0.05.*

**Table 2** shows the content of glucose, fructose and sucrose in globe artichoke’s offshoots, summer squash greens, faba greens and crenate broomrape. The highest glucose content was found in both types of globe artichoke’s offshoots (about 791 mg 100 g^-1^ FW on average). In comparison to these unconventional vegetables, the glucose content in crenate broomrape was about 40% lower, while in both summer squash greens and faba greens the glucose content (147 mg 100 g^-1^ FW on average) was about 81% lower. Fructose content in crenate broomrape was about 3.2-fold higher respect to offshoots of *Locale di Mola* globe artichoke and about sevenfold higher compared with other unconventional vegetables. As regards the sucrose content, the highest value was found in faba greens, while no significant differences were found between other unconventional vegetables.

**FIGURE 7:**
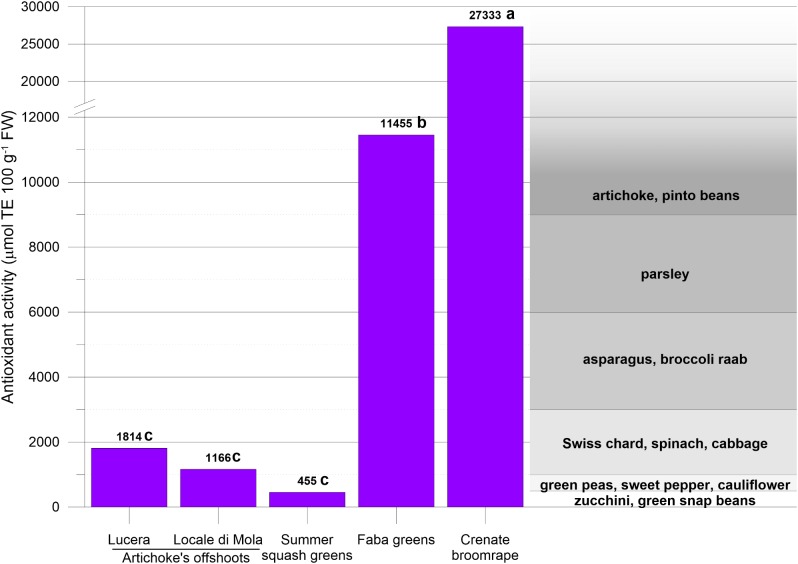
Antioxidant activity of globe artichoke’s offshoots, summer squash greens, faba greens and crenate broomrape, expressed as micromoles of Trolox Equivalent (TE) per 100 g of fresh weight (FW). Letters adjacent to data labels indicate statistical differences among the unconventional vegetables. The same letters indicate that mean values are not significantly different (*P* = 0.05). Typical antioxidant activity ranges for conventional vegetables are also reported according to Pennington and Fisher (2009).

The antioxidant activity of globe artichoke’s offshoots, summer squash greens, faba greens and crenate broomrape is reported in **Figure 7**, expressed in μmol Trolox equivalents (TE) 100 g^-1^ FW. Summer squash greens showed the lowest value followed by offshoots of globe artichokes. Instead, the highest value was found in samples of crenate broomrape.

## Discussion

Since offshoots of globe artichoke can be used in several traditional dishes like for recipes based on cardoons and heads of globe artichoke, it could be interesting to compare the proximate composition between these conventional and unconventional vegetables. Offshoots of both *Lucera* and *Locale di Mola* globe artichoke showed lower water content respect to cardoons but similar to heads of globe artichoke (**Tables 1**, **3**). With respect to the content of protein and carbohydrates, offshoots of globe artichoke showed lower content respect to heads of globe artichoke but higher respect to cardoons (**Tables 1**, **3**). The fiber content in offshoots of globe artichokes represents about 58 and 63% of total carbohydrate, respectively, for *Locale di Mola* and *Lucera* landraces (**Table 1**). It is also interesting to highlight that the AOAC Method 991.43 partially measures inulin and does not measure at all fructo-oligosaccharides (both prebiotic fiber). On the other hand, significant inulin contents were detected in offshoots. Therefore, actual total dietary fiber levels in offshoots are higher than those measured by this official method (Prosky and Hoebregs, 1999; Lattanzio et al., 2009). Thus, offshoots of globe artichokes may be considered a useful food to maintain bowel regularity in the short term, and to potentially afford a protection against chronic diseases in the long term. This because all the fibers of globe artichoke’s offshoots as a whole (prebiotic and other ones) can contribute directly to fecal bulking and indirectly, by stimulating the increase of probiotic microbial biomass (Flamm et al., 2001). Regarding the content of main cations (**Table 2**) offshoots of globe artichoke may be considered as low contributors to the sodium supply especially for *Lucera* landrace. Effectively, 100 g of this landrace of globe artichoke’s offshoots supplies about 3.2% of the Na daily intake (1.5 g per day) (European Food Safety Authority, 2006). As for *Locale di Mola* landrace, the same serving size of globe artichoke’s offshoots supplies about 10.7% of the daily intake. The higher sodium content in offshoots of *Locale di Mola* globe artichoke is probably due to proximity of the cultivated fields to sea (**Figure 2**) and therefore to the use of brackish water coming from aquifers. Moreover, the higher sodium content may have affected the potassium content considering the lower K content in *Locale di Mola* offshoots respect *Lucera* ones as well as the Na/K inverse correlation (**Table 2**), since sodium can substitute the K in the plant osmotic regulation. Anyway, both types of globe artichoke’s offshoots showed a higher sodium content respect to other unconventional vegetables (**Table 2**) confirming that plants belonging to the *Asteraceae* generally show a higher Na content in comparison with other botanic familes (Bianco et al., 1998). As for potassium, the results of the present study suggest that offshoots of *Locale di Mola* globe artichoke can be considered as low contributors to the daily supply of this cation. Considering an amount of 4.7 g K per day as an adequate intake (Food and Nutritional Board, 2005), 100 g of this unconventional vegetable could satisfy only about the 4% of this intake. Regarding the nitrate content (**Table 2**), offshoots of *Locale di Mola* globe artichoke may be considered as vegetables with a middle content (500–1000 mg kg^-1^ FW) like for cabbage (*Brassica oleracea* L. var. *capitata* [L.] DC.), dill (*Anethum graveolens* L.), radicchio (*Cichorium intybus* L., group *Rubifolium*), turnip (*Brassica rapa* L. subsp. *rapa* Thell.) and broccoli raab (*Brassica rapa* L. subsp. *sylvestris* L. Janch. var. *esculenta* Hort.), while offshoots of *Lucera* globe artichoke may be considered as vegetables with a relatively high content (1000–2500 mg kg^-1^ FW) like for celeriac (*Apium graveolens* L. group *Rapaceum*), fennel (*Foeniculum vulgare* Mill. var. *azoricum*), endive (*Cichorium endivia* L., group *Crispum*) and leek (*Allium porrum* L.) (Santamaria, 2006). For both *Lucera* and *Locale di Mola* landrace, the nitrate content in the offshoots is higher with respect to the heads of globe artichoke, which may be considered as vegetables with a very low content (<200 mg kg^-1^ FW) of this anion. This because NO_3_^-^ content differs in the various parts of a plant and its content in the stem (i.e., offshoots of globe artichoke) is higher respect to inflorescences (Santamaria, 2006). Regarding the sugar content, it is interesting to highlight that offshoots of globe artichoke show a similar total amount for both *Lucera* and *Locale di Mola* landrace as well as a similar composition of the different type of sugars. In fact, glucose represents the most abundant sugar, accounting for about 60 and 75% of the total amount, respectively, for *Lucera* and *Locale di Mola* landrace (**Table 2**). Therefore, these results suggest that the sugar content in offshoots of globe artichoke is scarcely influenced by genetic and environmental factors. The antioxidant activity measured in artichoke offshoots by DPPH assay was of about 1500 μmol TE 100 g^-1^ FW, showing comparable levels to those reported for other leafy vegetables by Pennington and Fisher (2009). The same authors assigned artichoke (inflorescence) to the vegetable class with antioxidant activity higher than 9000 μmol TE 100 g^-1^ FW. Differences in antioxidant activity between offshoots and inflorescences of artichoke are a probable consequences of their different morphological and maturity traits. However, the results of the present study are comparable with the antioxidant activity of cardoon leaf stalks, as showed by Lahoz et al. (2011), who reported values in the range 2.63–12.12 meq Trolox 100 g^-1^ for eight cardoon (*C. cardunculus* L. var. *altilis* DC) cultivars.

**Table 3:** Proximate composition of cardoon, globe artichoke, leafy chicory, Swiss chard, spinach, and asparagus.

	Cardoons	Globe artichoke	Leafy chicory	Swiss chard	Spinach	Asparagus
Water	94.00	84.94	92.00	92.66	91.40	93.22
Protein	0.70	3.27	1.70	1.80	2.86	2.20
Total lipid	0.10	0.15	0.30	0.20	0.39	0.12
Total carbohydrate	4.07	10.51	4.70	3.74	3.63	3.88
Fiber, total dietary	1.06	5.40	0.70	1.60	2.20	2.10


*Values are reported from the National Nutrient Database of the United States Department of Agriculture (United States Department of Agriculture [USDA], 2017).*

Summer squash greens may be used in several Italian recipes like for other conventional leafy vegetables such as chicory and Swiss chard. Thus, it could be interesting to compare the proximate composition of these conventional vegetables with that of summer squash greens. The water content is relatively higher in summer squash greens respect to both chicory and Swiss chard. On the other hand, the content of protein, lipid and carbohydrate results lower in summer squash greens respect to both chicory and Swiss chard, while the content of dietary fiber is higher in summer squash greens (**Tables 1**, **3**). Moreover, it is important to highlight that summer squash greens show a relatively low content of sugars considering also that about 50% of the total amount is represented by fructose (**Table 2**), having a very low glycemic index. Therefore, considering both content and composition of all types of carbohydrates (including fiber), summer squash greens could be recommended as a vegetable to use especially in the case of hypoglycemic diets. Regarding the content of principal cations (**Table 2**) summer squash greens may be considered as very low contributors to the sodium supply, since a serving size of 100 g supplies about 1.1% of the Na daily intake (European Food Safety Authority, 2006). With regard to nitrate content (**Table 2**), summer squash greens can be considered as a vegetables with a very low content (<200 mg kg^-1^ FW) like for summer squash fruits as well as other vegetables such as green beans (*Phaseolus vulgaris* L.), melons (*C. melo* L.), tomatoes (*Solanum lycopersicum* L.), peppers (*Capsicum annuum* L.) and potatoes (*Solanum tuberosum* L.) (Santamaria, 2006). It is very important to highlight that to the best of our knowledge there are no other leafy and/or leafy-like vegetables with a similar low NO_3_^-^ content. Therefore, these results suggest that summer squash greens should be recommended as a leafy vegetables to use especially for children’s, since European Regulation 1258/2011 (European Commission, 2011) imposes a maximum nitrate concentration of 200 mg 100 kg^-1^ FW for children food products.

Similarly to what reported for summer squash greens, also faba greens are traditionally used in Puglia for different recipes including first courses and side dishes. Considering that the similarity of the faba greens to spinach leaves (*Spinacia oleracea* L.) has been a decisive factor in using this plant part as a food, it could be interesting to compare the proximate composition between these conventional and unconventional vegetables. Faba greens showed a lower water content respect to spinach leaves but a higher protein content (about threefold higher) and total carbohydrate including fiber (**Tables 1**, **3**). Regarding the content of principal cations (**Table 2**) faba greens may be considered as very low contributors to the sodium supply, since a serving size of 100 g supplies about 1.4% of the daily intake (European Food Safety Authority, 2006). Regarding nitrate (**Table 2**) faba greens may be considered as vegetables with a middle content (range of 500–1000 mg kg^-1^ FW), similar to that observed in offshoots of *Locale di Mola* globe artichoke and other conventional vegetables (Santamaria, 2006). Instead, spinach is considered a vegetable with very high content of nitrate, since it contains more than 2500 mg NO_3_^-^⋅kg^-1^ FW (Santamaria, 2006). It is important to highlight that faba greens show also an interesting antioxidant activity, considering that a serving size of 100 g supply about 11,455 μmol of Trolox Equivalent (**Figure 7**). Therefore, the results of the present study suggest that faba greens may be considered as a very nutritious vegetable as well as a potential spinach substitute especially for its lower content of nitrate. Moreover, considering the use of faba greens also as a raw vegetable in salads (**Figure 5C**), it is possible to hypothesize the use of this unconventional vegetable also as a potential substitute of other vegetables with high NO_3_^-^ content such as lettuce and rocket (Santamaria, 2006).

Because the resemblance of the young stems of *O. crenata* L. to large asparagus was a decisive factor in eating this parasitic plant, it could be interesting to compare the proximate composition between asparagus and crenate broomrape. The water content is lower in crenate broomrape, while the content of protein and lipid and fiber is relatively similar between the two vegetables. On the other hand, crenate broomrape shows a content of total carbohydrate about threefold higher respect to asparagus (**Tables 1**, **3**). Regarding the content of principal cations (**Table 2**) like for summer squash greens and faba greens, also crenate broomrape may be considered as a very low contributor to the sodium supply, since a serving size of 100 g supplies about 1.4% of the daily intake (European Food Safety Authority, 2006). As for potassium, the results of the present study suggest that crenate broomrape may be considered as a low contributor to the daily supply of this cation, since 100 g of this unconventional vegetable could satisfy only about the 5.4% of daily adequate intake (Food and Nutritional Board, 2005). Regarding nitrate (**Table 2**) crenate broomrape may be considered a vegetable with a low-middle content (about 500 mg NO_3_^-^ kg^-1^ FW) according to the classification proposed by Santamaria (2006), though NO_3_^-^ content in crenate broomrape results higher respect to asparagus (Santamaria, 2006). It is important to underline that crenate broomrape shows a very interesting antioxidant activity, since a serving size of 100 g supply about 27,333 μmol of Trolox Equivalent (**Figure 7**). All these results suggest that crenate broomrape may be considered as a very nutritious Traditional Agri-Food Product of Puglia.

Finally, exploitation of offshoots of globe artichoke, summer squash greens, faba greens and crenate broomrape could be carried out through several initiatives, they integrating into the potential multi-functionality of farms. Thus, the exploitation of these unconventional vegetables may require a multi-disciplinary approach and integrated projects for protecting farmers communities and promoting the artisanal agri-food products. For example, the obtainment of labeled products such as Protected Designation of Origin (PDO), Protected Geographical Indication (PGI) and Traditional Specialties Guaranteed (TSG) could be a good opportunity.

## Conclusion

Offshoots of globe artichoke, summer squash greens, faba greens, and crenate broomrape have good potential as novel foods, being nutritious and refined products. Therefore, the exploitation of these unconventional vegetables may satisfy the needs of specific markets characterized by a demand for local and sustainable food products. On the other hand, the culinary use of these unconventional vegetables as well as their interesting quality traits are known only in some local areas. Therefore, specific activities are needed in order to disseminate knowledge, promote quality and boost consumer demand. Finally, offshoots of globe artichoke, summer squash greens and crenate broomrape require a long time before to be used (especially cleaning-up and soaking) and this may dissuade potential consumers. Therefore, new research should be carried out for obtaining new potential ready-to-eat retail products such as cook-chilled vegetables that may be an innovative and appealing idea for consumers.

## Author Contributions

MR: substantial contributions to the conception or design of the work, drafting the work, final approval of the version to be published, and agreement to be accountable for all aspects of the work in ensuring that questions related to the accuracy or integrity of any part of the work are appropriately investigated and resolved. AS: revised the article critically, final approval of the version to be published, and agreement to be accountable for all aspects of the work in ensuring that questions related to the accuracy or integrity of any part of the work are appropriately investigated and resolved. VP: performed the proximate analysis, interpretation of data, Revised the article critically, and final approval of the version to be published. PS: substantial contributions to the conception or design of the work, analysis and interpretation of data, final approval of the version to be published, and agreement to be accountable for all aspects of the work in ensuring that questions related to the accuracy or integrity of any part of the work are appropriately investigated and resolved.

## Conflict of Interest Statement

The authors declare that the research was conducted in the absence of any commercial or financial relationships that could be construed as a potential conflict of interest. The reviewer SP and handling Editor declared their shared affiliation.
